# Targeting PI3K/Akt signal transduction for cancer therapy

**DOI:** 10.1038/s41392-021-00828-5

**Published:** 2021-12-16

**Authors:** Yan He, Miao Miao Sun, Guo Geng Zhang, Jing Yang, Kui Sheng Chen, Wen Wen Xu, Bin Li

**Affiliations:** 1 MOE Key Laboratory of Tumor Molecular Biology and Key Laboratory of Functional Protein Research of Guangdong Higher Education Institutes, Institute of Life and Health Engineering, College of Life Science and Technology, Jinan University, Guangzhou, China; 2 Department of Pathology, The First Affiliated Hospital of Zhengzhou University, Henan Key Laboratory of Tumor Pathology, Zhengzhou, China; 3 MOE Key Laboratory of Tumor Molecular Biology and Guangdong Provincial Key Laboratory of Bioengineering Medicine, National Engineering Research Center of Genetic Medicine, Institute of Biomedicine, College of Life Science and Technology, Jinan University, Guangzhou, China

**Keywords:** Oncogenes, Drug development

## Abstract

The phosphatidylinositol 3-kinase (PI3K)/Akt pathway plays a crucial role in various cellular processes and is aberrantly activated in cancers, contributing to the occurrence and progression of tumors. Examining the upstream and downstream nodes of this pathway could allow full elucidation of its function. Based on accumulating evidence, strategies targeting major components of the pathway might provide new insights for cancer drug discovery. Researchers have explored the use of some inhibitors targeting this pathway to block survival pathways. However, because oncogenic PI3K pathway activation occurs through various mechanisms, the clinical efficacies of these inhibitors are limited. Moreover, pathway activation is accompanied by the development of therapeutic resistance. Therefore, strategies involving pathway inhibitors and other cancer treatments in combination might solve the therapeutic dilemma. In this review, we discuss the roles of the PI3K/Akt pathway in various cancer phenotypes, review the current statuses of different PI3K/Akt inhibitors, and introduce combination therapies consisting of signaling inhibitors and conventional cancer therapies. The information presented herein suggests that cascading inhibitors of the PI3K/Akt signaling pathway, either alone or in combination with other therapies, are the most effective treatment strategy for cancer.

## Introduction

The phosphoinositide 3-kinase (PI3K)/Akt signaling pathway is a major signaling pathway in various types of cancer.^1^ It controls hallmarks of cancer, including cell survival, metastasis and metabolism. The PI3K/Akt pathway also plays essential roles in the tumor environment, functioning in angiogenesis and inflammatory factor recruitment. The PI3K/Akt pathway can be aberrantly activated through various mechanisms, including different genomic alterations, such as mutations of PIK3CA, phosphatase and tensin homolog (PTEN), Akt, TSC1, and mechanistic target of rapamycin (mTOR).^2^ PI3K phosphorylates phosphatidylinositol-4,5-bisphosphate (PIP2) to generate phosphatidylinositol-3,4,5-trisphosphate (PIP3), and PIP3 then recruits oncogenic signaling proteins, including the serine and threonine kinase Akt.^3^ Once active, Akt phosphorylates a number of substrates. mTOR, one of the most common downstream effectors of Akt, integrates many proteins to promote cancer progression. Members of the PI3K/Akt/mTOR pathway are often mutated and activated in cancer.^4,5^

The study of PI3K/Akt networks has led to the discovery of inhibitors for one or more nodes in the network, and the discovery of effective inhibitors is important for improving the survival of patients with cancer. To date, many inhibitors of the PI3K/Akt signaling pathway have been developed, some of which have been approved for the treatment of patients with cancer in the clinic. However, many issues associated with the use of pathway inhibitors, including which drugs should be used to treat specific types of cancer and whether combination therapies will improve treatment outcomes, remain to be resolved. Current research aims to learn from clinical successes and failures to improve the design of clinical therapies based on pathway inhibitors and ultimately improve clinical cancer treatment.^6^

## Overview of the PI3K/Akt signaling pathway

### PI3Ks

Three classes of PI3Ks, class I, class II, and class III, have been identified, each of which has specific substrates and distinct effectors in addition to the common substrate Akt (Table 1).^3^ To date, class I, class II and class III PI3Ks have been widely mentioned in former studies.^7,8^ Further understanding the PI3K isoforms will help to fully elucidate the biological processes in various types of cancer cells.

**Table 1:** Different effectors of class I, II and III PI3Ks

PI3K isoforms	Effectors		References
Class I PI3K	Serine/Threonine kinases of the AGC kinase family	AKT	^183^
		PKC	^2^
		PDK-1	^183^
		SGK1	^244^
	Tyrosine kinases of the TEC family	BTK	^15^
		BMX	^245^
		ITK	^246^
	Guanine nucleotide exchange factors (GEFs)	Rac	^247^
		P-Rex1	^16^
		Tiam1	^248^
		Vav1	^249^
		Vav2	^249^
Class II PI3K		Sortins	^250^
		Small GTPase	^251^
		Myotubularins	^252^
Class III PI3K		LKB1	^26^
		SGK3	^253^

#### Class I PI3Ks

Class I PI3Ks, heterodimers that consist of a p110 catalytic subunit and a p85 regulatory subunit, exert their functions by activating downstream tyrosine kinases, such as G protein-coupled receptors (GPCRs) and small monomeric GTPases.^9^ Moreover, the p85 subunit can transmit various cellular signals by laying a critical foundation for signal integration and the activation of downstream proteins.^10^

Class I PI3Ks are divided into four catalytic isoforms, p110α, p110β, p110γ and p110δ, and they are encoded by PIK3CA, PIK3CB, PIK3CG and PIK3CD, respectively.^3^ Among the four class I catalytic isoforms, PIK3CA is mentioned commonly in human cancer due to its frequent mutations.^6^ The time point of pathway activation varies among cancer types and patients. For example, the activation of PIK3CA mutations is an early event in breast and colon cancer.^11^ In contrast to PIK3CA, transforming mutations in the PIK3CB gene are rare; however, the gene is ubiquitously expressed, probably due to the distinct mode of interaction between this isoform and regulatory subunits. PIK3CD is mainly expressed in white blood cells and B cells and is indispensable for B cell follicle maturation and survival.^12^ Although the PIK3CG level is related to cancer growth, reduced PIK3CG expression has been reported to promote colon cancer growth and development.^13^ According to differences in the regulatory subunits, class I PI3Ks are further divided into class IA and class IB enzymes, and both of their catalytic subunits are formed by the PI3K catalytic core and extended N-terminally with the Ras-binding domain (RBD).^9,14^ In cancer, somatic genetic activation through multiple mechanisms is very common in the class IA PI3K pathway. These mechanisms include inactivation of PTEN and the p110 catalytic subunit. Activating mutations of the p110 catalytic subunits often occur in PIK3CA, while mutations in PIK3CB and PIK3CD are much less frequent.^4,14^

Class I PI3Ks play an important role in the development of cancers and exert their functions by regulating downstream effectors. The downstream effectors of class I PI3Ks share a pleckstrin homology (PH) domain and include serine/threonine kinases from the AGC kinase family, tyrosine kinases expressed in hepatocellular carcinoma (HCC, TEC family) and guanine nucleotide exchange factors (GEFs). Akt is also a member of the serine/threonine AGC kinase family, which will be introduced in detail below. TEC family members are key effectors of class I PI3Ks in lymphocytes. As a member of the TEC family, Bruton tyrosine kinase (BTK) has a highly selective PH domain for PIP3. Activation of the BTK signaling pathway triggers the growth of B cell malignancies. BTK is often overexpressed in chronic lymphocytic leukemia (CLL) cells, and its phosphorylation level is increased.^15^ GEFs are also important effectors of class I PI3Ks. PI3K activates and promotes Rac-mediated actin recombination in cancer- and growth factor-stimulated fibroblasts. In addition to modulating cell morphology and motility, this PI3K/Rac signaling axis drives an increase in glycolysis flow by releasing aldolase from actin filaments.^16^

#### Class II PI3Ks

Class II PI3Ks comprise the C2α, C2β, and C2γ catalytic isoforms and lack regulatory subunits; thus, they can be activated as monomers.^17^ In mammals, three class II PI3K isoforms have been identified, among which PI3KC2α and PI3KC2β are broadly expressed, while PI3KC2γ is mainly expressed in the liver.^18^ PI3KC2α plays a pivotal role in breast cancer progression by affecting mitotic spindle formation.^19^ In addition, class II PI3Ks contain additional protein-binding domains and an extended N-terminal region, which contributes to intracellular localization. Another feature of class II PI3Ks is that they do not produce PIP3 in vitro; however, they can generate PIP2 using PIP as a substrate, which is significantly different from the functions of class I and III PI3Ks.^20^

Because the lipid products generated by class II and class I PI3Ks are substantially different, class II PI3Ks may activate different downstream effectors.^21^ Sortins, small GTPases and myotubularins are three main downstream effectors of class II PI3Ks.^22^ Additionally, the catalytic pocket of class II PI3Ks differs from that of class I and III PI3Ks. Currently, class II PI3Ks function as major signaling enzymes and play important roles under normal and pathological conditions.^22^ These three isoforms of class II PI3Ks importantly contribute to various cellular activities because they synthesize unique lipid products in cancer.^23^

#### Class III PI3Ks

Class III PI3K VPS34 (also called PIK3C3) is unique, as it plays an important role in regulating autophagy and macrophage phagocytosis by binding to a protein complex composed of a regulatory subunit and a catalytic subunit. Therefore, heterodimeric class III PI3K can also regulate autophagy.^7^

The smallest PI3K catalytic core is the constituent element of VPS34, and it forms tetrameric complex I and tetrameric complex II.^7^ Complex I play a vital role in the formation and extension of autophagosomes (wrapping and separating cytoplasmic components), mainly by promoting recruitment at the phagocytosis initiation site in the endoplasmic reticulum after activation. Complex II has the advantage of increasing autophagosome-late endosome/lysosome fusion by controlling endosome maturation.^24^

While VPS34 does not directly regulate signal transduction, it transduces signals by regulating various protein kinases when activated by amino acids. For example, in breast cancer treatment, the use of Akt inhibitors upregulates VPS34-dependent SGK3 signaling.^25^ Another kinase regulated by VPS34 is intimal LKB1 liver kinase B1 (LKB1; also known as STK11), an AMPK activator and a positive regulator of cell polarity and epithelial tissue.^26^ VPS34 and mTOR complex 1 (mTORC1) signaling have been shown to be correlated. VPS34 exerts its function by activating acute stimuli but not by controlling the basal activity of mTORC1.^27^ Therefore, approaches targeting VPS34 may be a useful clinical treatment strategy. Although the widely used VPS34 inhibitor 3-methyladenine is not selective, related studies have documented the use of a highly selective inhibitor of VPS34.^28^

### Akt

The serine and threonine kinase Akt, also known as protein kinase B (PKB), was discovered 25 years ago.^29^ The upstream and downstream targets of Akt have also been studied. Various diseases are induced by Akt dysfunction, including cancer.^25,30^ Three Akt isoforms have been identified in mammals: Akt1, Akt2 and Akt3. Akt1 and Akt2 are enriched in many tissues, such as pancreatic tissue, while Akt3 is mainly expressed in the brain, and its expression is very limited by its tissue distribution.^31^ Different Akt isoforms play different and vital roles in cancer; for example, Akt2 is involved in cancer cell migration and invasion, and Akt3 is associated with hormone independence.^31,32^ Moreover, Akt2 gene expression is amplified in pancreatic cancer, while Akt3 is overexpressed in breast and prostate cancer, and both Akt2 and Akt3 are insensitive to hormones.^33^

Akt is classically activated by receptor tyrosine kinases (RTKs) and GPCRs,^3^ and activated Akt recruits and activates one or more subtypes of class I PI3Ks on the plasma membrane. In turn, activated PI3K also phosphorylates the T ring and the C hydrophobic motif, two key residues in the core activation domain of Akt1, which regulate the corresponding residues of Akt2 and Akt3.^34^ In addition, IGF1 activates Akt. When IGF1 binds to IGF1R, IRS-1 and PI3K are recruited and activated. Activated PI3K converts PIP2 to PIP3, which activates phosphoinositide-dependent kinase-1 (PDK1) and then influences Akt.^35^ TRAF6 and TBK1 also modulate the activity of Akt.^36^ In conclusion, Akt activation is easily monitored, and research on class I PI3K signal transduction might thus be performed by focusing on Akt.

## Major upstream activators of the PI3K/Akt signaling pathway

**Fig. 1:**
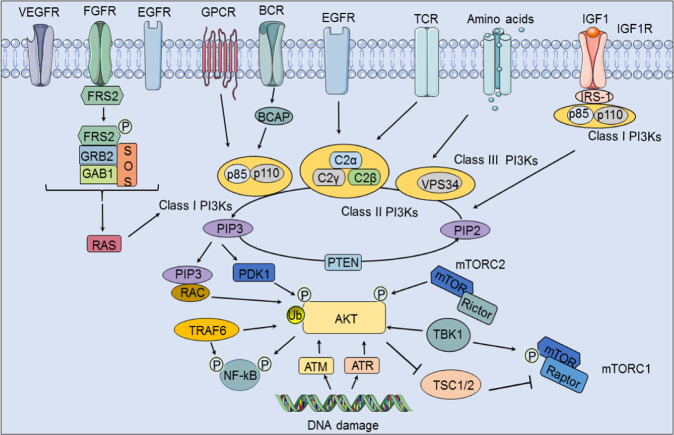
Upstream activation of the PI3K/Akt signaling pathway. On the one hand, ligands combined with specific RTKs (EGFR, VEGFR and FGFR) can activate class I PI3Ks via RAS; on the other hand, class I PI3Ks can be activated by BCRs through B cell adapters and by GPCRs. The FGFR substrate FRS2 is phosphorylated in combination with GRB2, SOS and GAB1 to activate class I PI3Ks. In addition to being activated by EGFR, class II PI3Ks can be activated by TCRs. While class III PI3Ks are activated by amino acids, total activated PI3K phosphorylates the third carbon of the PIP2 inositol head and transforms it into PIP3 to thereby activate AKT via PDK1 and RAC, and this transformation process can be inhibited by PTEN. In addition, IGF-1 in combination with IGF1R can recruit IRS-1 and class I PI3Ks and then participate in the conversion of PIP2 to PIP3. Moreover, mTORC2 can affect the activity of Akt by affecting the phosphorylation of Akt and then affect downstream mTORC1 via TSC1/2, and both Akt and mTORC1 can be activated by TBK1. Moreover, TRAF6 can affect the activity of Akt by affecting its ubiquitylation. Furthermore, DNA damage can affect Akt via ATM and ATR

Upstream activation of the PI3K/Akt signaling pathway is essential for its function in cancer and other related diseases. Activation of this pathway is related to many factors, including the RTK family, Toll-like receptors (TLRs), and B-cell antigen receptors (BCRs). Major upstream components of the PI3K/Akt signaling pathway are discussed below (Fig. 1).

### RTKs

RTKs constitute a transmembrane protein family with intrinsic phosphotyrosine kinase activity that mainly includes epidermal growth factor receptors (EGFRs), vascular endothelial growth factor receptors (VEGFRs) and fibroblast growth factor receptors (FGFRs). RTKs remain inactive in the plasma membrane prior to their activation by ligands.^37^ Ligands such as homologous growth factors, cytokines, and hormones activate PI3K signaling pathways by activating RTKs.^38^ Furthermore, the p85 subunit of class I PI3Ks binds to phosphorylated RTK, resulting in a conformational change in the catalytic domain of PI3K (p110).

#### EGFRs

EGFRs are 170-kDa RTKs that phosphorylate tyrosine residues and activate class I PI3K and class II PI3K signal transduction by binding to ligands and forming homodimers or heterodimers.^39,40^ EGFRs belong to the RTK ErbB family, which also includes ErbB-3, ErbB-4, and HER2. HER2 forms HER2/EGFR heterodimers with EGFR, and HER2/EGFR heterodimers have greater signal transduction potential than EGFR homodimers.^41^ Inhibitors designed based on this feature have obvious effects on clinical efficacy and may delay drug resistance. The other family member, ErbB-3, has six tyrosine phosphorylation sites and effectively binds to PI3K.^42^ The ligand TGF-α also binds to EGFRs, activates the PI3K signaling pathway and promotes tumor growth and metastasis in colorectal cancer cells.^43^ EGFR antibodies, such as cetuximab and panitumumab, inhibit PI3K/Akt signal transduction by competitively binding to EGFRs, thus inhibiting the occurrence and development of cancer.^44^

#### VEGFRs

VEGFRs are RTKs that are categorized three main types: VEGFR-1 (Flt-1), VEGFR-2 (KDR, Flk-1) and VEGFR-3 (Flt-4). Among the three types, VEGFR-1 and VEGFR-2 mainly function in endothelial cells, while VEGFR-3 is present in the lymphatic endothelium.^45^ The VEGFR extracellular domain binds to VEGF, leading to the dimerization and phosphorylation of the intracellular tyrosine kinase domain and to the activation of downstream proteins. Therefore, VEGFRs vitally stabilize neovascularization and promote cell survival and migration.^46^ Similarly, VEGF forms VEGF/VEGFR-2 dimers, which activate the PI3K/Akt pathway to mediate tumor metastasis and angiogenesis.

VEGFR-2-mediated activation of the PI3K/Akt signaling pathway is important for tumor survival. The binding of a ligand to VEGFR-2 activates PI3K and phosphorylates PIP2, resulting in the accumulation and reactivation of PIP3 to thereby activate the PI3K/Akt signaling pathway. VEGFR inhibitors used in combination with PI3K/Akt/mTOR signaling pathway inhibitors represent an effective therapeutic strategy.^47^ Furthermore, VEGFR-1 and VEGFR-2 were shown to interact using the siRNA method. The siRNA-mediated knockout of VEGFR-1 in endothelial cells resulted in the attenuation of VEGFR-2 promoter activity, suggesting that VEGFR-1 plays an important role in inducing and modulating multiple signaling pathways.^48^

#### FGFRs

FGFRs are very important for cancer metastasis and angiogenesis. For example, FGFR amplification in breast cancer affects the phosphorylation of downstream fibroblast growth factor receptor substrate 2 (FRS2), resulting in PI3K/Akt signaling pathway activation.^49^ High FGFR levels lead to PI3K/Akt pathway activation, which is involved in tumor growth. Further studies revealed that changes downstream of the FGFR pathway affect tumor cells and alter the tumor microenvironment.^50^ In addition, the inhibition of FGFRs may result in gene reprogramming.^51^

In summary, RTKs are involved in PI3K/Akt pathway activation. Mutations in RTKs potentially result in ligand-independent transduction, which can be suppressed by pathway inhibitors in non-small-cell lung cancer (NSCLC).^52^ The activation of class IA PI3Ks is related to RTK signaling, and p85 regulatory subunits are essential for the RTK-mediated activation of class IA PI3Ks. Therefore, RTK inhibitors might negatively regulate PI3K signaling.^53^ However, many types of cancer are always resistant to a single RTK inhibitor because PI3K is activated by multiple RTKs in cancer.^54^ Therefore, understanding RTK signaling networks will facilitate a better comprehension of the PI3K pathway and further exploration of cancer therapies.

### TLRs

TLRs are expressed in immune and nonimmune cells and are important in cancer progression.^55,56^ TLRs can identify conserved microbial motifs in bacterial lipopolysaccharides (recognized by TLR4) and single- or double-stranded RNA (identified by TLR3).^57^ TLRs also distinguish and bind endogenous ligands, thereby activating the signaling pathway.^58^ For example, some studies found that natural TLR4 ligands activate Akt by increasing its phosphorylation in a time-dependent manner, which promotes the progression of colorectal cancer.^59^ Similarly, activated TLR4 protects cells from chemotherapy, leading to drug resistance in head and neck squamous cell carcinoma.^60^ TLR3 activation leads to cancer cell apoptosis and has antitumor effects on renal cell carcinoma and melanoma.^61^

### BCRs

The signaling pathways activated by BCRs are crucial for the development, activation, and differentiation of B cells.^62^ Among them, the PI3K/Akt pathway is particularly important. In B cells, class I PI3Ks are activated by BCRs via the B-cell receptor associated protein (BCAP), which is an important step in PIP3 production and Akt activation.^63^ BCRs and cytoplasmic adapters profoundly affect PI3K/Akt signaling pathway activation, and Akt is not activated when B cells lack BCRs.^64^

The noncatalytic region of the tyrosine kinase (NCK) family comprises a set of BCR adapters that are recruited to BCR signaling complexes, which are essential factors that activate B cells to exert their function. The NCK structure is characterized by three SRC homology 3 domains at the N-terminus and an SH2 domain at the C-terminus.^65^ NCK affects PI3K/Akt signaling in B cells, and although Akt phosphorylation is decreased to some extent in the absence of NCK1, almost no phosphorylation occurs when both NCK1 and NCK2 are deficient.^63^

To date, BCR research has mainly focused on CLL. According to previous studies, BCR signaling is vital for the maintenance of cancer cell survival, and its function is downregulated by p110δ or the inhibition of BTK.^66^

### GPCRs

GPCRs constitute the largest cell surface protein family and play an important role in cell signal transduction; additionally, they are a common target of the PI3K/Akt signaling pathway.^67^ GPCRs transmit signals through heterotrimeric G proteins and regulate downstream effector pathways by interacting with various small G proteins that bind directly to GPCRs and participate in the regulation of signaling networks. One characteristic of GPCRs is that they recognize and respond to chemically distinct ligands to effectively activate PI3K/Akt signaling in different cells.^3,68^ Moreover, GPCRs regulate cancer cell proliferation and survival, and their persistent activation affects mitotic and metabolic responses, which are the basis for tumorigenesis.

The predominant mechanisms by which GPCRs activate PI3K are tissue-specific, and many GPCR ligands, such as sphingosine 1-phosphate, activate PI3Ks.^69^ GPCRs activate PI3K/Akt signaling by stimulating Ras to thereby activate class I PI3Ks, which regulate cancer and many other diseases. Small GTPases also promote tumor metastasis by controlling PI3K/Akt signaling.^70^

### PTEN

PTEN is a tumor suppressor that is key for maintaining normal physiological activity; PTEN was initially identified as a gene prone to mutations in multiple types of sporadic tumors.^71^ The C2 domain houses the PTEN lipid substrate and is necessary for the proper localization of PTEN on the plasma membrane; PTEN has shown affinity for the phospholipid membrane in vitro.^72^ Nuclear PTEN is important for the maintenance of chromosomal integrity and centromere stability.^73^

As a lipid phosphatase, PTEN negatively regulates the PI3K signaling pathway and transforms PIP3 into PIP2.^74^ When PTEN is mutated or participates in another form of inactivation, PI3K effectors, especially Akt, become activated without any exogenous oncogenic stimulus.^75^ In particular, PI3K phosphorylates membrane-bound PIP2 to generate PIP3. The binding of PIP3 to the PH domain anchors Akt on the plasma membrane and allows it to be phosphorylated and activated by PDK1. Usually, PTEN participates in tumor signal transduction by dephosphorylating protein targets such as focal adhesion kinase (FAK), insulin receptor substrate 1, c-SRC, and PTEN itself.^76^ The overactivation of Akt by PTEN is the most important carcinogenic factor in PTEN-deficient cancers. In addition, PTEN plays an important role in the control of tumor cell migration and angiogenesis.^77^

## Major effectors downstream of the PI3K/Akt signaling pathway

**Fig. 2:**
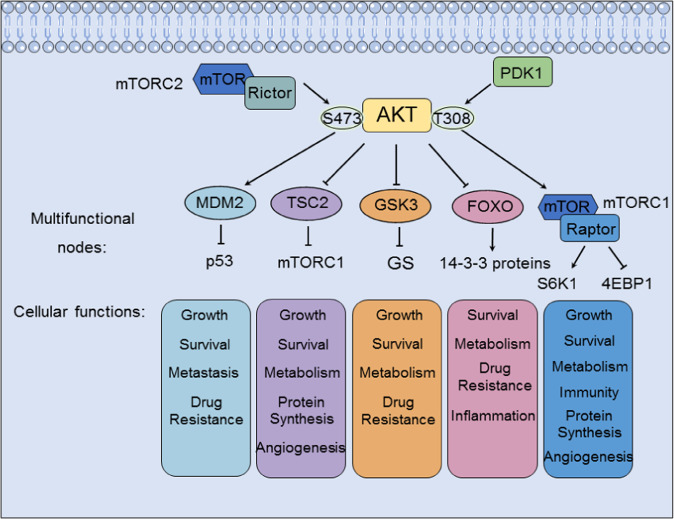
Downstream effectors of the PI3K/Akt signaling pathway and their cellular functions. The activation of Akt signaling can promote (arrows) or inhibit (blocking arrows) the phosphorylation of downstream effectors. Downstream regulation by Akt contributes to many cellular processes, including tumor growth, tumor survival, tumor cell proliferation, cancer immunity, cancer metabolism and cancer angiogenesis

Akt phosphorylates downstream effectors on serine and threonine in a sequence-dependent manner, which typically recognizes substrates containing the consensus phosphorylation motif R-X-R-X-X-S/T.^78^ Akt signaling promotes tumor cell survival, proliferation, growth, and metabolism by activating its downstream effectors. Here, we introduce the major targets downstream of the PI3K/Akt signaling pathway (Fig. 2).

### mTOR

More than 100 Akt substrates have been discovered, although not all of them have been confirmed.^78^ Due to its parallel regulation of different substrates, the downstream effects of Akt signaling are extensive. mTOR is reported to regulate tumor growth, survival, metabolism, and immunity. As a protein kinase, mTOR is considered an atypical member of the PI3K-related kinase family, and it is usually assembled into complexes, such as mTORC1 and mTOR complex 2 (mTORC2), to play critical roles in many biological processes.^79^

Many growth factors and their receptors, such as VEGF and VEGFR, act as positive regulators to transmit signals to mTOR through the PI3K/Akt pathway.^80^ Under normal conditions, PI3K activity is at a basal level. After stimulation with growth factors, signals are transmitted to PI3K. Then, PI3K catalyzes the production of PIP3, which binds to the PH domain of Akt. This step is limited by a negative regulator of mTOR named PTEN.^81^ Akt and mTOR are thought to interact, as Akt was shown to activate mTOR through the phosphorylation of tuberous sclerosis complex 2 (TSC2).^82^

The mTORC1 complex is composed of mTOR, mLST8, raptor, and PRAS40. mTORC1 controls cell growth in part by phosphorylating S6 kinase 1 (S6K1) and eIF-4E-binding protein 1 (4EBP1), known regulators of protein synthesis.^83^ Furthermore, mTORC1-mediated signaling to HIF1α and LIPIN1 increases glucose metabolism and lipid synthesis, respectively.^84^ mTORC2 is composed of mTOR, mLST8, SIN1 and rictor. Activated mTOR interacts with its protein subunits and forms mTORC2.^85^ Akt is phosphorylated by mTORC2 upon activation by growth factors. In conclusion, mTOR inhibition has enormous potential in clinical cancer therapy.

### GSK3

The multifunctional serine and threonine protein kinase glycogen synthase kinase 3 (GSK3) was the first reported Akt substrate.^86^ Two subtypes of GSK3, GSH3α and GSK3β, have been identified. These two subtypes share 85% sequence homology, and they were originally identified to be associated with the glycogen synthesis response to insulin.^87^ Studies have confirmed that different GSK3 subtypes have specific functions in different tissues.^88^

GSK3 is considered to be expressed at the crossroads of multiple biochemical pathways in some diseases.^89^ The EGFR/RAS/PI3K/PTEN/Akt/GSK3/mTORC1 pathway is common in cancer, and GSK3 is one of its targets. After the Akt-induced phosphorylation of either Ser21 (α) or Ser9 (β) in N-terminal regulatory domains in response to PI3K-mediated signaling, GSK3 (both GSH3α and GSK3β) is inactivated and targeted for proteasomal degradation.^86,90^ The Akt-mediated phosphorylation of GSK3 produces an intramolecular pseudosubstrate that blocks the binding pocket and inhibits the substrate from approaching GSK3.^91^ GSK3 expression affects various biochemical processes in cancer. In addition to tumor growth, GSK3 participates in tumor metabolism by phosphorylating and inhibiting metabolic enzymes such as its substrate glycogen synthase (GS).^92^ Similarly, another study suggested that the GSK3 inhibitor IX increases apoptosis and alters the structures of membrane lipids.^93^

### FOXOs

Forkhead box Os (FOXOs) are a subgroup of a forkhead box (FOX)-containing transcription factor (TF) superfamily. FOXO TFs include four direct downstream targets of Akt: FOXO1, FOXO3, FOXO4, and FOXO6. In mammals, these TFs control the expression of many target genes^94^ and are expressed in specific tissues. FOXO1 and FOXO4 are predominantly expressed in adipose tissue and skeletal muscle, respectively, whereas FOXO3 tends to be expressed in the brain, heart, kidney and spleen. In contrast, FOXO6 is expressed mainly in the adult brain, suggesting its important function in the nervous system.^95^

A major characteristic of FOXOs is the strict control of their localization in the cytoplasm and nucleus. FOXOs contain a nuclear localization signal (NLS) domain and a nuclear export signal (NES) domain, which closely regulate FOXO shuttling.^96^ A study found that the PI3K/Akt signaling pathway partially regulates cell survival by phosphorylating FOXOs to thereby increase their binding to the 14-3-3 protein, which masks the NLS (also blocking nuclear translocation from the cytoplasm but increasing FOXO removal from the nucleus) and leads to the final ubiquitin proteasome pathway (UPP)-dependent degradation of FOXOs.^97^ Therefore, the insulin/PI3K/Akt pathway is essential for adjusting FOXO levels by phosphorylation.

Similarly, the highly conserved genetic relationship between Akt and FOXOs supports the hypothesis of an important regulatory association existing between Akt and FOXO suppression. This association was first identified in *C*. elegans, in which the loss of FOXO family members rescued dauer-stage arrest caused by the depletion of Akt-1 and Akt-2.^98^ The activity and substrates of Akt are regulated by the phosphorylation of threonine 308 and serine 473,^99^ and the phosphorylation of serine 473 is not essential for the Akt-mediated phosphorylation of TSC2 and GSK3β; however, it is essential for the phosphorylation and inactivation of FOXOs.^100^ In summary, these biochemical and genetic studies have confirmed that the main phenotypes induced by Akt depletion are driven by FOXO-mediated transcription; therefore, FOXOs are downstream targets of Akt signaling in various biological reactions.

### TSC2

PI3K and Akt stimulation by growth factors such as IGF1 is an evolutionarily conserved function that promotes cell growth through the Akt-mediated activation of mTORC1 and the subsequent phosphorylation and inhibition of TSC2 (also known as tuberin).^82^ TSC1 and TSC2 are encoded by tumor suppressor genes that are mutated in tuberous sclerosis (TSC).^101^ The complex comprising TSC1 and TSC2 suppresses mTORC1 activity; through a C-terminal domain and a GTPase-activating protein domain, TSC2 converts Ras-related Rheb-GTP, a potent activator of mTORC1, to Rheb-GDP,^102^ thereby inactivating mTORC1. However, the Akt-mediated phosphorylation of TSC2 reverses this process and is thus important for the regulation of mTORC1 and might impair the ability of TSC2 to inhibit Rheb and mTORC1.

### MDM2

Decreased p53 levels have been proposed to arrest the cell cycle, while increased p53 levels induce cell apoptosis.^103^ The activation of PI3K/Akt signaling may be induced by the suppression of p53 through the activation of another tumor promoter, MDM2.^104^ MDM2 is an oncogene that induces tumorigenesis, and its mRNA level is controlled by p53 in response to oxidative stress and DNA damage.^105^ These findings, in addition to the finding that MDM2 forms a complex with wild-type p53, demonstrate that MDM2 exerts its oncogenic function by interacting with wild-type p53 and suppressing the transcription of target genes.^106^

The intracellular localization of MDM2 is posttranslationally modulated by PI3K/Akt.^107^ A study revealed that PI3K/Akt phosphorylates a serine in the MDM2 domain.^108^ Because phosphorylation is essential for the transfer of MDM2 from the cytoplasm to the nucleus, activated PI3K/Akt can induce the nuclear translocation of MDM2, bypass mitogen stimulation, and activate PI3K/Akt signaling. Therefore, after nuclear entry, MDM2 binds to the tumor suppressor p53, inhibits its transcription, and induces its degradation.^108,109^ Consistently, the suppression of the PI3K/Akt pathway by PTEN protects p53 from MDM2-induced degradation. Furthermore, p53 expression is positively associated with DNA damage.^110^

## Crosstalk with other pathways

According to previous studies, no pathways exist independently. Other signaling pathways are associated with the PI3K/Akt pathway network through direct regulation or downstream targets.

### Crosstalk with MAPK signaling

The RAS/RAF/MEK/ERK (MAPK) pathway is important in cellular processes such as tumor cell proliferation, survival and invasion. The mutation of components such as RAS, RAF and MEK results in dysregulation of the pathway in various types of cancer.^111^ Moreover, 30% of RAS GTPases are activated by mutations in these types of cancer, but inhibitors that directly target RAS proteins have not been developed. To date, research has aimed to develop inhibitors of the downstream RAF/MEK/ERK and PI3K/Akt pathways.^112^

According to reports, the PI3K/Akt and MEK/ERK pathways cooperate in tumor growth.^113^ Signaling pathways also interact with each other, and the enhancement of one signaling pathway may enhance or inhibit another pathway. For instance, the effect of PI3K/mTOR inhibitors on tumor cells is blocked by the inhibition of MEK or knockout of ERK, and the combined inhibition of MEK and PI3K/Akt/mTOR signaling thus inhibits tumor cell growth.^114^ In addition, MAPK pathway signals function as second messengers to attenuate PI3K/Akt signals by decreasing reactive oxygen species (ROS) levels. MEK1/2 inhibition upregulates Akt phosphorylation and MEK1/2 inhibition in response to mild hypoxia.^115^ These effects may provide negative feedback for the PI3K/Akt-induced activation of MAPK pathways.

### Crosstalk with NF-κB signaling

The NF-κB TF family includes components such as p50 and p65; NF-κB is a heterodimer that is isolated in the cytoplasm by inhibitor of kappa B (IκB).^116^ Upon the phosphorylation of IκB, NF-κB is released, enabling its nuclear translocation and binding to genes involved in processes such as cell proliferation and angiogenesis in esophageal cancer.^117^ Some studies have suggested that Akt signaling phosphorylates IκB kinase α to activate NF-κB TFs, which are downstream of multiple signaling pathways.^118^

A regulatory circuit between the EGFR/PI3K/Akt/mTORC1 and IKK/NF-κB signaling pathways has been identified in cancer.^119^ The EGFR/PI3K/Akt/mTORC1 signaling pathway regulates the IKK/NF-κB signaling pathway, while IKK/NF-κB also regulates EGFR expression and subsequently modulates the PI3K/Akt pathway. Inhibitors targeting IKK effectively block the EGFR/PI3K/Akt and IKK/NF-κB signaling pathways, which is very important for the use of IKK inhibitors as a single drug or in combination with other inhibitors in clinical trials.^119^

### Crosstalk with Wnt/β-catenin signaling

Although the Wnt/β-catenin and PI3K/Akt pathways have different carcinogenic mechanisms, they have been shown to be associated. The Wnt pathway is an important factor maintaining intestinal homeostasis; it regulates the self-renewal of stem cells and increases the proliferation of intestinal epithelial cells, and overactivation of the Wnt pathway may eventually lead to cancer.^120,121^ Some studies have shown an association between the Wnt/β-catenin and PI3K/Akt pathways in cancer. Wnt/β-catenin pathway activation is mediated by phospholipase D1PLD1 (PLD1), which downregulates ICAT via the PI3K/Akt signaling axis.^122^

Moreover, in breast cancer, activation of the PI3K/Akt pathway by Nectin-4 induces the activation of the Wnt pathway and then affects the proliferation of tumor stem cells, which is an important mechanism by which cancer stem cells achieve self-renewal.^123^ Communication between the Wnt/β-catenin and PI3K/Akt pathways has been observed in different types of human cancer, and PI3K/Akt pathway activation leads to Wnt/β-catenin pathway inhibition. In contrast, when the PI3K/Akt pathway is inhibited, the Wnt/β-catenin pathway is overactivated.^124^

## PI3K/Akt signaling and cancer

### PI3K/Akt signaling and tumorigenesis

At present, tumors are generally thought to be propagated by somatic cells, constituting the tumorigenesis process. Due to dysregulation of their self-detection function, cells are unable to identify their own mutations and “quit” dividing on time and instead replicate and reproduce with mutations, resulting in the accumulation of mutations. Moreover, autoimmune deficiencies, endocrine disorders, and other adverse stimuli also provide conditions that support the process of tumorigenesis. A study identified AMPK as a vital regulator of Akt activation by various stresses in tumorigenesis.^125^ Other studies have also shown that the PI3K/Akt signaling pathway regulates its downstream effectors, thereby promoting the occurrence of tumors.^126^

As mentioned above, FOXO is a vital target protein in the PI3K/Akt signaling pathway. After phosphorylation by PI3K/Akt signaling, the entry of FOXO into the nucleus is blocked,^127^ which prevents the expression of its target genes and eliminates its transcriptional effects on AR, ERG, Runx2, and other target genes, thereby promoting the occurrence of cancer.

Based on the important role of the PI3K/Akt signaling pathway in tumorigenesis, some oncogenes in the PI3K/Akt signaling pathway are positively regulated. For example, KDM5a plays a vital role in the occurrence of tumors. It promotes the formation of HCC lesions by regulating miR-433-FXYD3-PI3K-Akt signaling.^128^ Therefore, studies on this pathway and its related pathways may be crucial to increase the anticancer efficacies of clinical PI3K/Akt inhibitors.

### PI3K/Akt signaling and tumor growth

One important feature of cancer cells is their uncontrolled proliferation, and the proliferation rate determines the type of tumor therapy. EGFRs regulate the proliferation of tumor cells through signal transduction pathways. Studies have shown that the PI3K/Akt signaling pathway affects the cell cycle by modulating its downstream targets, thereby promoting the proliferation of tumor cells.^129^

Akt phosphorylates cyclin-dependent kinase inhibitors and prevents p27 from translocating to the nucleus, thereby weakening its inhibitory effect on the cell cycle and directly promoting the proliferation of tumor cells.^129^ Akt also promotes the proliferation of tumor cells through its downstream effector p27. Since the PI3K/Akt signaling pathway can directly control the proliferation of tumor cells, some proteins are also involved in cancer cell proliferation through the PI3K/Akt pathway. Furthermore, insulin growth factor 2 (IGF2) is involved in the development of many malignancies and alters the cancer cell proliferation process by regulating the PI3K/Akt pathway.^130^

During cancer treatment, the PI3K/Akt signaling pathway is often targeted to inhibit cancer cell proliferation; for example, the signaling pathway is controlled by the inhibition of lncRNA TDRG1 expression in osteosarcoma cells, thus interfering with their proliferation.^131^

### PI3K/Akt signaling and apoptosis

Apoptosis is an autonomous cell death process.^132^ Abnormal apoptosis and uncontrolled growth allow cancer cells to rapidly proliferate. PI3K/Akt signaling blocks the expression of proapoptotic proteins, reduces tissue apoptosis and increases the survival rate of cancer cells.^133^

Akt inhibits the proapoptotic factors Bad and procaspase-9 through phosphorylation and induces the expression of the proapoptotic factor Fas ligand. In addition, Akt activation is associated with resistance to increased apoptosis induced by tumor necrosis factor (TNF)-associated apoptosis-inducing ligand (TRAIL/APO-2L), a member of the TNF superfamily that has been shown to have selective antitumor activity.^134^ Similarly, Akt negatively regulates the function or expression of Bcl-2 homology domain 3-only proteins, which play a proapoptotic role by inactivating the original prosurvival Bcl-2 family members. In conclusion, inhibition of the PI3K/Akt signaling pathway, which has been shown to regulate cancer cell apoptosis, can serve as a new direction for future research on cancer treatment.^131^

### PI3K/Akt signaling and drug resistance

In tumor therapy, cancer drug resistance is the main reason for treatment failure and indirectly promotes tumor progression.^135^ The dysregulation of PI3K/Akt signaling also plays an important role in cancer drug resistance. Furthermore, studies have demonstrated that the IGF1R/p110β/AKT/mTOR axis confers resistance to BYL-719 in PIK3CA mutant breast cancers.^136^ Similarly, a study showed that targeting PI3K/Akt signaling pathway components can be used to overcome drug resistance in cancer therapy.^137^

#### Chemotherapy resistance

Chemotherapies are mainly used to destroy tumor cell DNA and thereby prevent the cells from replicating, eventually affecting cell survival. During routine chemotherapy, no treatment interval exists, allowing resistant cells to be generated and leading to tumor regeneration.^138^ In addition, DNA destruction is prevented in drug-resistant cells due to their dormancy.^139^

The PI3K/Akt signaling pathway is important for the drug resistance of different types of cancer, such as lung cancer^140^ and esophageal cancer.^141^ PI3K/Akt inhibitors inhibit tumor growth and induce tumor cell apoptosis. For NSCLC cells with high Akt expression, the use of PI3K/Akt signaling pathway inhibitors increases their cell apoptosis induced by chemotherapy and reduces their resistance to chemotherapy; furthermore, inhibition of the PI3K/Akt signaling pathway effectively improves drug-induced lung cancer cell apoptosis.^142^ Moreover, members of the PI3K/Akt signaling pathway play an important role in antiestrogen resistance in breast cancer.^143^

#### Immunotherapeutic resistance

The discovery and application of immune checkpoint inhibitors have substantially advanced the treatment of malignant tumors. Thus far, CTLA-4, PD-1 and PD-L1 have achieved significant clinical efficacy and have been approved for the treatment of many types of cancer.^144^ However, the problem of immune drug resistance persists. Some patients do not respond to immunotherapy, while other relapse after immunotherapeutic treatment. The PI3K/Akt signaling pathway plays an important role in the regulation of immune checkpoints and sensitivity to immune checkpoint inhibitors. Studies have shown that the activation of the Akt signaling pathway caused by the deletion of PI3KCA mutations is strongly related to the upregulation of PD-L1 expression in the prostate gland.^145^ In mouse lung cancer models and human lung cancer cell lines, the Akt signaling pathway regulates the expression of PD-L1 at the protein translation level.^146^ In conclusion, the PI3K/Akt signaling pathway is obviously related to immune resistance, which provides a basis for combined therapeutic strategies including immune checkpoint inhibitors and PI3K pathway inhibitors in the future.

### PI3K/Akt signaling and cancer metabolism

One of the main features of cancer is the occurrence of metabolic aberrations, such as changes in glycolytic pathway. Metabolic aberrations will lead to changes in signaling pathways, affecting the occurrence, development and metastasis of cancer. Under physiological conditions, the PI3K/Akt signaling pathway is activated by the actions of insulin, growth factors and cytokines to regulate metabolism in organisms.^147^ In cancer cells, the activation of oncogenes in the PI3K/Akt signaling pathway reprograms cellular metabolism by enhancing the activities of nutrient transporters and metabolic enzymes, thus supporting the anabolic needs of abnormally growing cells.^147^

PI3K/Akt signaling not only regulates metabolism-associated proteins such as SREBP and alters metabolism through phosphorylation mediated by metabolic enzymes but also indirectly alters metabolism by controlling various TFs.^147^ The phosphorylation of metabolic enzymes causes acute changes in the activities of metabolic pathways and the directionality of metabolic fluxes, while long-term changes in cellular metabolism are usually achieved by controlling gene expression programs.^147^ Akt not only directly phosphorylates a variety of metabolic enzymes and nutrient transport regulators but also regulates cellular metabolism by activating mTORC1, GSK3 and FOXO.^91^ The phosphorylation of TSC2 by Akt leads to Rheb-GTP aggregation, which in turn activates mTORC1 to thereby enhance glucose metabolism and lipid synthesis.^84^ GSK3, a key regulatory factor in cellular metabolism, participates in cellular metabolism by phosphorylating and inhibiting metabolic enzymes, such as its substrate glycogen synthase.^86,87,92^ FOXO TFs regulate cellular metabolism and tumor suppression.^148^ Akt phosphorylates FOXO and prevents it from entering the nucleus to mediate the expression of its target genes.^127,149^ In addition, accumulating evidence indicates that PI(3,4)P2 is not only a waste product for the removal of PI(3,4,5)P3 but also functions as a signaling molecule in cancer metabolism.^150^

### PI3K/Akt signaling and angiogenesis

Angiogenesis in tumors mainly refers to the growth of new capillaries from the existing capillary network that supply tumor cells with nutrition. Among the three subtypes of Akt, Akt1 is the main subtype that regulates the normal physiological function of endothelial cells. After activation by VEGF, Akt promotes the proliferation, migration and survival of endothelial cells, thus affecting angiogenesis.^151^ This finding also provides lateral support for the conclusion that endothelial nitric oxide carbon synthase (eNOS), which controls vascular tone, is a specific substrate of Akt1 in endothelial cells.^152^ At the same time, the loss of Akt1 in mouse endothelial cells reduces the amount of nitric oxide on the cell membrane and affects the formation of blood vessels.^153^ Akt1 is also related to vascular remodeling, which is mediated by endothelial cells. Expression of the activated Akt1 allele prevents the formation of intimal lesions after arterial injury, induces pathological angiogenesis, and increases vascular permeability.^154^

In addition, a recent report revealed that the p110α subtype of PI3K promotes tumor angiogenesis in homogenic mouse models and that the inactivation of p110α leads to an increased vascular density, decreased vessel size and altered pericyte coverage on vessels. This decrease in vascular function is associated with increased tumor necrosis and decreased tumor growth. The role of p110α in tumor angiogenesis is mediated by several factors, such as the regulation of endothelial cell proliferation and the expression of delta-like protein 4 (DLL4). Thus, p110α regulates angiogenesis in the tumor stroma, and inactivation of p110α suppresses tumor angiogenesis and tumor growth.^155^

### PI3K/Akt signaling and cancer metastasis

Studies have shown that cancer metastasis is the main cause of poor prognosis.^156^ The mechanism of cancer metastasis involves many factors, such as genetic material, surface structure, invasion, adhesion, angiogenesis and lymphangiogenesis. Two main mechanisms by which PI3K/Akt signaling promotes cancer metastasis have been identified.

First, the PI3K pathway promotes metastasis by reducing intercellular adhesion and enhancing mobility. After extracellular signaling molecules bind to specific receptors on the surface of the cell membrane, they are activated by different signal transduction pathways in the cell to modulate the activities of different TFs, resulting in differential degrees of epithelial cell phenotype transformation. The PI3K/Akt signaling pathway plays an important role in the induction of squamous cancer cell epithelial-mesenchymal transition (EMT). PI3K activation produces the second messenger PIP3 downstream of Akt activation, which activates or inhibits downstream target proteins via phosphorylation, thereby regulating cell survival, proliferation, and differentiation as well as the composition of the cytoskeleton, among other processes, inducing EMT. PI3K/Akt signaling pathway activation increases tumor cell invasion and metastasis.^157^ Steelman et al. reported that the continuous activation and high expression of PI3K/Akt are closely related to EMT in NSCLC.^158^

Second, the PI3K pathway promotes metastasis by promoting tumor neovascularization, which is required for the metastatic spread of tumors. Multiple signaling pathways have been shown to be involved in the regulation of tumor angiogenesis, among which PI3K/Akt signaling is the most important.^159^ PI3K forms a complex with E-cadherin, β-catenin, and VEGFR-2 and is involved in endothelial signaling mediated by VEGF through the activation of the PI3K/Akt pathway.^160^ The PI3K/Akt signaling pathway also promotes TNF-induced endothelial cell migration and regulates tumor angiogenesis.^161^ Matrix metalloproteinases (MMPs) and cyclooxygenase 2 (COX-2) also affect tumor angiogenesis. In tumor invasion and metastasis, platelet-derived growth factor (PDGF) induces MMP expression through a PI3K-mediated signaling pathway.^162^ Upregulation of the antiapoptotic protein Bcl-2 and activation of the PI3K/Akt signaling pathway are the main mechanisms by which COX-2 stimulates endothelial angiogenesis.^163^

However, not all components of the PI3K/Akt signaling pathway promote metastasis. One of the Akt isoforms, Akt1, can exert an antimetastatic effect, which is accompanied by increased ERα expression.^164^ Breast cancer cells undergo differentiation upon the activation of Akt1, thereby losing their metastatic potential.^165^ Similarly, Akt1 can inhibit metastasis in mice by regulating MMP9 and E-cadherin.^166^

### PI3K/Akt signaling and inflammation

Virchow first proposed an association between inflammation and cancer in 1863,^167^ and many subsequent studies have supported this association.^168^ A study showed that the PI3K/Akt signaling pathway promotes the development of inflammation by affecting neutrophils, lymphocytes and other white blood cells.^169^ However, IL-1, IL-6, TFN-α and other inflammatory factors activate Akt and expand the range of inflammation, while Akt inhibition blocks both inflammation and tumor development.^170^ IL-6 activates the PI3K/Akt signaling pathway to promote the expression of B-FGF, which induces angiogenesis.^171^ mTOR and p70 S6K1, downstream effectors of Akt, directly act on endothelial cells to promote tumor inflammation.^172^

Studies have shown that the positive effect of Akt signaling on inflammatory cells is conducive to promoting the aggregation of reactive cells, and the resulting oxidative stress reaction leads to the accumulation and release of peroxides (ROS) at the tumor site.^173^ In addition, IL-37 is an anti-inflammatory cytokine that has been reported to induce autophagy and apoptosis by regulating PI3K/Akt/mTOR signaling.^174^

Inflammation also promotes cancer development by activating the PI3K/Akt signaling pathway. For example, inflammation-induced PI3K/Akt signaling regulates the permeability and migration of endothelial cells and affects cancer progression.^175^ In conclusion, inflammation affects the biological processes of endothelial cells in tumors through the PI3K/Akt signaling pathway, which is an important component of inflammation-induced tumor development.

### PI3K/Akt signaling and immunity

Immunity is divided into innate and adaptive immunity. The immune system plays an important role in tumor detection and elimination. To date, various immunotherapies have been applied in the clinic. The PI3K/Akt signaling pathway also exerts a vital effect on the immune system. Growth factors, cytokines, and other factors activate Akt signaling in myeloid cells, thus activating downstream effectors of Akt.

#### Innate immunity

The innate immune system, which is composed of monocytes, leukocytes and macrophages, is the first line of defense against infection.^176^ According to some studies, inhibition of the PI3K signaling pathway reduces the secretion of proinflammatory cytokines, and the PI3K pathway is related to the movement of macrophages.^177^

The PI3K signaling pathway affects the secretion of proinflammatory cytokines from innate immune system cells by regulating the activity of downstream targets. It participates in the movement and adhesion of macrophages and plays an important role in cells.^178^ On the other hand, Akt signaling is the key signal transduction pathway activated in macrophages under various external stimuli.^179^ Akt signaling is activated in both M1 and M2 macrophages, but most of the current evidence suggests that Akt signaling exacerbates the M2 state. Hence, interventions targeting PI3K signaling may be an effective treatment for some immune and tumor-related diseases.^180^ Similarly, the PI3K/Akt pathway affects natural killer (NK) cells. A study reported reduced NK cellularity and a decreased number of CD27(high) NK cells in mice expressing p110 δ (D910A), which is a catalytically inactive form of p110 δ, and that inactivated p110 δ slightly impaired NK-mediated tumor cell cytotoxicity in vitro and in vivo.^181^

#### Adaptive immunity

The adaptive immune system mainly includes T and B cells, each of which have unique antigen receptors. PI3K signaling plays a key role in T and B cells.^182^ PI3Kδ has been identified as the main subtype of PI3K activated by T cell receptors (TCRs) and BCRs, and activated PI3Kδ subsequently activates Akt and other downstream signals.^62^

TEC family proteins, such as BTK, play a key role in PI3K signal transduction.^183^ BTK inhibition reduces the output of the PI3K signal in B cells.^66,184^ Cooperation between the PI3K signaling pathway and BTK in immune cells has also been documented. Upon the antigen activation of B cells, the cells undergo cloning, expansion and differentiation and secrete different antigen-specific antibodies. Some B cells rapidly differentiate into plasma cells, mainly producing low-affinity IgM, while others secrete high-affinity conversion antibodies. This differentiation is determined in part by the levels of PI3K/Akt signaling and FOXO TF activity.^185^

In CD8+ T cells, class IA PI3Ks are mainly activated by RTKs, such as TCRs and cytokine receptors. Activated PI3K/Akt signaling promotes the uptake of glucose and amino acids by CD8+ T cells for energy-demanding cellular processes, such as proliferation and cytokine synthesis and secretion.^186^ Moreover, research has shown that Akt inhibition effectively enhances memory T cell differentiation in cancer.^187^ Therefore, inhibition of the PI3K/Akt signaling pathway may affect T cell function. The PI3K/Akt signaling pathway plays an indispensable role in the immune system.

### Functional role of the PI3K/Akt signaling pathway in the tumor microenvironment

The tumor microenvironment plays an important role in the occurrence, development, and metastasis of tumors and influences cancer treatment.^188^ The tumor microenvironment includes immune cells, endothelial cells, fibroblasts, the extracellular matrix, and signaling molecules surrounding tumor cells. PI3K/Akt and its downstream effectors are known to constitute a signaling pathway involved in tumor cell development. PI3K signals regulate cell survival, development, and proliferation by relying on extracellular signaling molecules, and signaling molecules outside tumor cells are part of the tumor microenvironment.

In the tumor microenvironment, cancer-associated fibroblasts (CAFs) are the most abundant cells in the tumor matrix and promote tumor progression by secreting several growth factors, such as FGFs.^189^ Hepatocyte growth factor (HGF) secreted by CAFs specifically triggers PI3K/Akt signaling to affect cancer progression, and HGF has been detected in many types of human cancer, such as ovarian cancer.^190^ Furthermore, CCL5 derived from CAFs has been detected in ovarian cancer cells and shown to influence drug resistance by regulating PI3K/Akt signaling.^191^

**Fig. 3:**
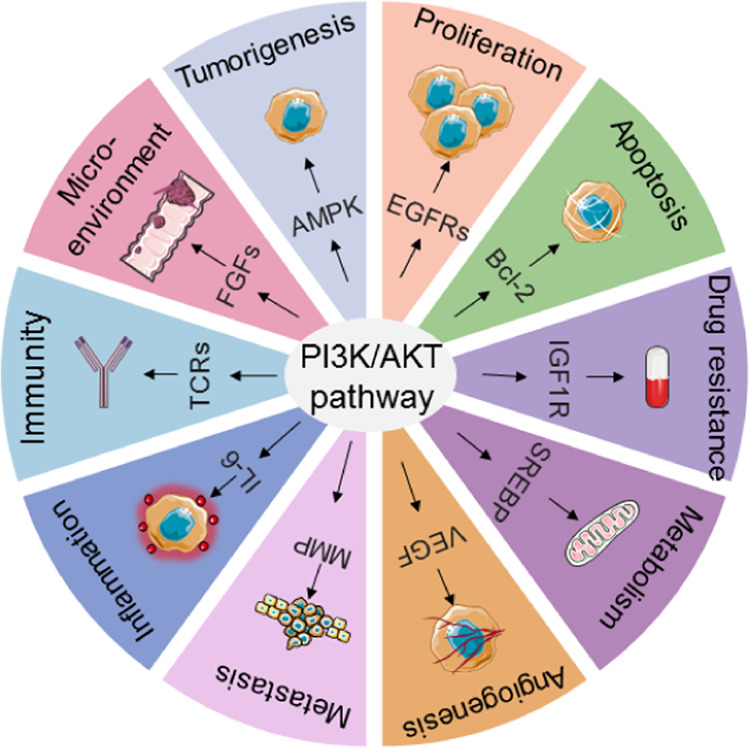
PI3K/Akt signaling and cancer. Various biological processes are regulated by the PI3K/Akt pathway via key mediators/pathways

In addition, tumor growth depends on oxygen and nutrients delivered by new blood vessels,^192^ which are in turn related to endothelial cells, an important component of the tumor microenvironment. As shown in previous studies, PI3K subtypes promote the differentiation of endothelial cells and other cells,^193^ and different PI3K inhibitors thus produce different vascular responses. Interventions targeting PI3K in CAFs and endothelial cells in the tumor microenvironment in combination with conventional therapies have great potential in the treatment of cancer. In summary, the PI3K/Akt signaling pathway plays an essential role in multiple cancer phenotypes, as summarized in Fig. 3.

## Cancer therapies targeting the PI3K/Akt pathway

### PI3K inhibitors

Because of the function of PI3K/Akt signaling in oncogenesis, various inhibitors targeting PI3K are being explored for the treatment of various types of cancer (Table 2); to date, some agents have been approved for the treatment of malignancies and for the improvement of cancer therapy.^194^

**Table 2:** Novel agents targeting PI3K signaling in cancer

Class	Inhibitor	Phase	Toxic effects	Reference
Pan‑PI3K inhibitors	GDC-0941	I	Neutropenia、Neuropsychiatric effects (confusion, depression, anxiety)、Hepatotoxicity、Diarrhea	^254^
	BKM-120	II	Hyperglycemia	^255^
	BAY-80-6946	Approved	Nausea High blood sugar	^256^
	XL-147	I	Rash	^257^
	PX-866	II	Diarrhea ALT/AST elevation	^258^
Isoform-selective PI3K inhibitors	BYL-719	I	Hyperglycemia	^259^
	GDC-0032	I	Rash、Diarrhea、Pneumonitis	^206^
	IPI-145	Approved	Hyperglycemia、Rash、Diarrhea、Hypertension	^260^
	SAR-260301	Preclinical	Nausea、Vomiting、Diarrhea	^261^
	MLN-1117	I	Nausea	^262^
Dual pan‑PI3K and mTOR inhibitors	BEZ-235	I/II	Fatigue/asthenia、Thrombocytopenia	^263^
	SF-1126	I/II	Hyperglycemia	^264^
	GDC-0980	I	Maculopapular rash Symptomatic hyperglycemia	^265^
	PF-04691502	II	Fatigue Loss of appetite、Nausea、High blood sugar、Rash、Vomiting	^266^
	XL-765	I	Stomatitis	^267^
	GSK-2126458	I	Diarrhea	^268^
	PF-05212384	II	Fatigue	^266^
	GSK-1059615	Preclinical	Immunosuppression	^269^
	BGT-226	I/II	Diarrhea Nausea Loss of appetite Vomiting Fatigue	^270^

#### Pan-PI3K inhibitors

Pan-PI3K inhibitors target the p110 subunits of class IA PI3Ks, which are the most widely involved subunits in cancer. These inhibitors have greater anticancer activity and fewer side effects (less toxicity) than inhibitors of other PI3K classes. Some representative small-molecule pan-PI3K inhibitors include pictilisib (GDC-0941), buparlisib (BKM120) and pilaralisib (XL147).^195^

GDC-0941, a thienopyrimidine derivative, was the first inhibitor to enter clinical trials. As a pan-class I PI3K inhibitor, GDC-0941 shows the same activity (IC50 = 3 nM) against p110α and p110δ enzymes, and in kinase assays, it exerts an inhibitory effect on p110β and p110γ at nanomolar concentrations.^196^ Experiments have shown that monotherapy or combination therapy with GDC-0941 and other agents has strong antitumor activity in mouse xenograft models of human cancer.^196,197^

BKM120, also known as NVP-BKM120, is another effective pan-class I PI3K inhibitor that exerts a strong inhibitory effect on p110-α and p110β enzymes, with IC50 values of 52 nM and 166 nM, respectively. It also effectively inhibits p110δ and p110γ enzymes, with IC50 values of 116 nM and 262 nM, respectively.^198^ Preclinical studies have shown that BKM120 hinders the growth of U-87MGGBM cells after xenotransplantation in the brain, which helps to prolong the xenotransplantation duration in host animals; most importantly, it does not induce significant adverse effects.^199^ According to a research report describing a phase I study of 35 patients with advanced solid tumors receiving BKM120 at 12.5–150 mg per day, the administration of BKM120 at the maximum tolerated dose of 100 mg/day was safe and exhibited preliminary antitumor activity.^200^

Another class I inhibitor, XL147, has high specificity for four subtypes of PI3Ks.^201^ Analysis of its selectivity for more than 130 protein kinases revealed that XL147 is much more selective for class I PI3Ks than other kinases. In cell experiments, XL147 inhibited the formation of PIP3 in the cell membrane and the phosphorylation of kinases such as Akt in a variety of tumor cell lines, influencing genetic alterations in the PI3K pathway.^201^ In mouse transplant models, the oral administration of XL147 inhibited the phosphorylation of Akt, p70S6K, and S6 for at least 24 h, and repeated administration of XL147 significantly inhibited tumor growth.^201^

#### Isoform-selective PI3K inhibitors

Compared to pan-PI3K inhibitors, isotype-specific inhibitors exhibit less off-target toxicity because they are specific to the tumor type; thus, isotype-specific inhibitors can be administered at higher doses and are more effective.^202^ Alpelisib (BYL-719) and taselisib (GDC-0032) are representative homospecific inhibitors.

BYL-719 is the first inhibitor to selectively target class I p110α. In a phase I trial, BYL-719 resulted in monotherapy sensitivity in patients with PI3KCA-mutant solid tumors.^203^ The combination of BYL-719 and fulvestrant resulted in the remission of 29% of patients with severe pretreated PI3KCA-mutant tumors and had a good safety profile in patients.^204^

Based on the antitumor activity of BYL-719 in phase I clinical trials, scientists conducted a phase III clinical trial of BYL-719 combined with fulvestrant in the treatment of advanced breast cancer.^205^ The phase III trial showed a significantly prolonged survival rate of patients with advanced breast cancer. The overall response of patients treated with BYL-719 plus fulvestrant was better than that of patients treated with the placebo plus fulvestrant. However, patients treated with BYL-719 plus fulvestrant had higher rates of hyperglycemia, rashes and diarrhea than those treated with the placebo.

GDC-0032 exhibits the same activity against p110α, p110γ and p110δ, but its inhibitory effect on p110β is 30-fold lower.^206^ Due to the stronger selectivity of GDC-0032, this drug has shown better efficacy in the treatment of PI3KCA-mutant tumors, with a response rate of 36% among breast cancer patients with PI3KCA-mutant tumors.^207^ In a phase III clinical trial, GDC-0032 was more effective than fulvestrant,^208^ and the results showed that the main adverse events after GDC-0032 administration were diarrhea and hyperglycemia. In some patients, the moderate improvement in progression-free survival after GDC-0032 administration occurred at the cost of significant toxicity, and GDC-0032 was thus not suitable for use in these patients. In terms of symptoms after administration, GDC-0032 produced more side effects than BYL-719, possibly because of its stronger specificity and exertion of a more obvious inhibitory effect on p110α.^206^

#### Dual pan-PI3K and mTOR inhibitors

Since both mTOR and PI3K have a p110 subunit with similar structures, treatment with dual pan-PI3K and mTOR inhibitors may exert an improved therapeutic effect due to the more efficient inhibition of the PI3K/Akt/mTOR signaling pathway. The current pan-PI3K and mTOR inhibitors mainly include SF1126, dactolisib (Bez235), voxtalisib (XL765) and GSK1059615.^30^ Although this type of inhibitor is less specific than isotype-specific inhibitors, it has the potential to treat a variety of tumors with a wide range of genetic abnormalities; however, it also has many unknown toxicities and side effects.

Another advantage of these inhibitors is that they completely inhibit the p110 subunit; however, they should not be used in conjunction with other PI3K and Akt inhibitors.^6^ Key factors affecting this class of inhibitors include whether the dose at which all p110 subunits are completely suppressed is tolerable in patients and whether the use of these inhibitors is at the expense of other inhibitor targets.^6^ mTORC1 inhibitors tend to activate the PI3K signaling pathway through feedback inhibition.^209^ Therefore, the advantages of dual pan-PI3K and mTOR inhibitors in inhibiting feedback inhibition are highlighted, and they have obvious therapeutic advantages.

### Akt inhibitors

Due to the existence of three Akt subunits, most current inhibitors of Akt are pan-Akt inhibitors,^74^ and the research and development of specific inhibitors against the three subunits of Akt will be very challenging. Several inhibitors of Akt are currently being investigated in clinical trials, such as MK-2206, GSK-2141795, GSK-2110183, AZD5363 and GDC0068 (Table 3).

**Table 3:** Current inhibitors targeting Akt signaling in cancer

Inhibitor	Phase	Target	ClinicalTrials.gov Identifier:	Reference
Perifosine	II	AKT1/2/3		^271^
GSK-690693	I	AKT1/2/3		^272^
VQD-002	I	AKT1/2/3		^273^
AZD-5363	III	AKT1/2/3	NCT04493853	
GDC-0068	III	AKT1/2/3	NCT04650581	
GSK-2141795	II	AKT1/2/3	NCT01964924	
M2698	I	AKT1/3		^274^
GSK-2110183	II	AKT1/2/3	NCT01531894	
MK-2206	II	AKT1/2/3	NCT01370070	

MK-2206, an allosteric pan-Akt inhibitor, shows synergistic activity with cytotoxic compounds such as doxorubicin, gemcitabine, docetaxel and carboplatin in lung cancer.^75^ In addition, MK-2206 inhibits the phosphorylation of Akt mediated by carboplatin and gemcitabine, thereby enhancing the therapeutic effect of the drug by inhibiting tumor survival laterally.^210^ MK-2206 also enhances erlotinib activity in erlotinib-sensitive patients and erlotinib-resistant NSCLC cell lines.^211^ Recently, MK-2206 entered preclinical studies to determine its effects on acute myeloid leukemia (AML). Data obtained from mouse models in preclinical studies indicate that weekly administration is effective for the treatment of AML, which supports its use in subsequent clinical trials.^212^ A preclinical study on the inhibitor MK-2206 in nasopharyngeal carcinoma (NPC) is also ongoing.^213^ HGF was used to resensitize drug-resistant cells to the drug, revealing the therapeutic efficacy of MK-2206.^211^ The efficacy of MK-2206 was also reported in a phase I clinical trial in which the tumors of patients with PTEN-deficient pancreatic cancer regressed after treatment with MK-2206 alone. MK-2206 also exerts a mild therapeutic effect on patients with melanoma and endocrine tumors.^214^

AZD5363 is an inhibitor targeting the kinase activity of the three Akt subtypes (Akt1, 2, and 3).^215^ AZD5363 inhibits cancer cell proliferation and phosphorylates GSK3β and the downstream channel protein S6 in vitro. In vivo, AZD5363 inhibits tumor growth in xenograft tumor models and maintains pharmacodynamic activity for at least 24 hours.^215^ Preclinical sensitivity to AZD5363 is strongly associated with the presence of PIK3CA, and this trend has also been observed for other inhibitors of the PI3K/Akt/mTOR pathway.^216^

### mTORC1 and mTORC2 inhibitors

Studies have shown that mTOR, a classical downstream effector of the PI3K oncogenic pathway, has activity ranging from 40-90% in various solid tumors.^217^ Therefore, the use of mTOR as a target for cancer treatment has become a research hotspot.

#### Rapamycin

Rapamycin and its analogs (temsirolimus, everolimus, and deforolimus) are allosteric inhibitors of mTOR and constitute the first generation of mTOR inhibitors.^218^ Rapamycin binds to mTOR and FKBP-12 to form a ternary complex and specifically inhibits the phosphorylation of the mTORC1 protein kinase S6K1, thus inhibiting mTORC1 activity.^218^ Rapamycin is produced by *Streptomyces sp*. and possesses antifungal properties. In the 1980s, rapamycin was reported to have anticancer activity, but its clinical application was limited due to its poor water solubility and stability.^218^

Although rapamycin inhibits mTOR with high specificity, its efficacy in different environments depends on its dosage. Clinical doses of rapamycin may vary due to the differential sensitivities of cancer cells to rapamycin. The amount of rapamycin required to phosphorylate the substrate also differs for different mTOR substrates, which is caused by the competitive inhibition of mTOR by phosphatidic acid and rapamycin.^219^

#### ATP-competitive mTOR inhibitors

Strategies involving the targeting of mTORC1 and mTORC2 have been developed to inhibit mTOR more completely, and many ATP-competitive mTOR inhibitors have been exploited. ATP-competitive mTOR inhibitors are a class of small-molecule ATP analogs that compete with ATP to occupy mTOR kinase active sites.^218^ Unlike rapamycin analogs, these ATP analogs ensure the complete blockade of mTORC1 and mTORC2, thereby preventing Akt phosphorylation caused by mTORC2 and the observed resistance to rapamycin analogs. In vitro studies have shown that ATP-competitive inhibitors exert a greater inhibitory effect than rapamycin analogs.^218^ AZD8055, one of the most recent and potent ATP-competitive mTOR inhibitors, functions by inhibiting the phosphorylation of Akt and its downstream proteins and has been shown to induce autophagy in cancer cells and inhibit tumor growth in vivo.^220^ In conclusion, inhibitors targeting the PI3K/Akt pathway are promising for cancer therapy, and numerous PI3K/Akt pathway inhibitors have been developed (summarized in Table 4).

**Table 4:** Therapies targeting PI3K/Akt signaling

Agent	Targets	Inhibitors
Apoptosis	p53	BKM120
		CAL-101
		GDC0068
	p-S6	BYL719
		GDC-0068
		Rapamycin
	p38	GSK2141795
Proliferation	p53	Bez235
		Temsirolimus
	p-S6	XL765
		XL147
		GSK2121183
		LY2780301
		Rapamycin
		Deforolimus
	CDK1	Everolimus
	TGF-β1	Bez235
Autophagy	LC3B	MK2206
	p-S6	AZD5363

### Combination therapeutic strategies

Cancer treatment has always been difficult and perplexed humans for many years, and combination therapy is an inevitable trend. A logical approach is to combine PI3K-Akt pathway inhibitors with standard targeted drugs approved for the treatment of specific malignancies. This approach will also facilitate the inclusion of PI3K/Akt inhibitors in treatment regimens for patients with early-stage disease and preclude their use for only patients with relapsed or refractory tumors. Here, we describe the combination of PI3K signaling inhibitors with growth factor inhibitors, MAPK inhibitors, and conventional therapies, such as chemotherapy, radiation therapy and immunotherapy.

#### Combination with GFR inhibitors

GFR alterations are some of the most common causes of cancer among various types of tumors. For example, abnormal HER2 amplification is common in breast cancer.^221^ GFR inhibition has several advantages for tumor therapy, as it not only blocks signal initiation and crosstalk with complementary pathways but also increases the therapeutic sensitization of tumor cells to chemotherapy and radiation.^221^ A trial of buparlisib in combination with paclitaxel and trastuzumab for breast cancer with abnormal HER2 amplification showed that buparlisib can be used in combination with paclitaxel and trastuzumab.^222^ Additionally, alpelisib in combination with cetuximab can be used for the treatment of head and neck squamous cell carcinoma.^223^

#### Combination with MAPK inhibitors

The PI3K and MAPK signaling networks have been shown to interact, which creates a potential pathway for the development of combination therapies (PI3K and MAPK pathway inhibitors) for cancer. Additionally, complete inhibition of the PI3K and MAPK signaling pathways is required for tumor control.^224^ The results of a clinical trial on treatment with both PI3K and MAPK signaling inhibitors support this hypothesis, as PI3K and MAPK therapies used in combination improved the treatment efficacy compared to that achieved with either inhibitor alone. However, the disadvantage of this approach is increased toxicity.^225^

In a phase I trial, GDC-0941 and SAR245409 were used to target PI3K in combination with GDC-0973 and AS-703026 to target MEK and showed promising results in the treatment of KRAS-mutated tumors.^226,227^ Further research on combinations of drugs targeting these two signaling pathways is imperative. PI3K and MAPK inhibitor combination therapies should include solid tumors that depend on MAPK pathway activation, such as BRAF-mutant and KRAS-mutant melanoma, colorectal carcinoma and ovarian cancer.^226–228^

#### Combination with chemotherapy

According to previous studies, PI3K signaling pathway inhibitors may increase the sensitivity of tumor cells to chemotherapy by changing the peripheral vascular system and tumor perfusion, thereby increasing the efficacy of systemic therapy and inducing apoptosis.^229^ Early clinical studies showed that PI3K pathway inhibitors are well tolerated when administered with chemotherapy; pictilisib, carboplatin, and paclitaxel have shown good antitumor activity in patients with NSCLC. Additionally, a phase II clinical trial on ipatasertib was initiated because of its positive effect on gastric cancer when used in combination with chemotherapy.^230^

According to recent studies, PI3K signaling inhibitors increase DNA damage and sensitize cell lines to poly (ADP-ribose) polymerase inhibitors.^231^ Therefore, treatments targeting the association between the PI3K pathway and DNA repair are emerging as a therapeutic strategy for BRCA1-deficient tumors. A phase I trial on buparlisib in combination with olaparib is underway in patients with triple-negative breast cancer and highly serous ovarian cancer.^232^ The results of these trials might further our understanding of this combination and provide new insights into strategies for overcoming resistance.

#### Combination with radiotherapy

Since the 21st century, radiation therapy for cancer has been continuously improved, as the damage to normal patient tissue has been reduced, allowing the therapeutic dose to be increased, and the cure rate among patients with cancer has been improved.^233^ Despite the improvements in radiation therapy for cancer treatment, the survival rate of patients with squamous cell carcinoma of the head and neck in the last five years was only 40%.^234^ With combination therapy, inhibitors of the PI3K/Akt signaling pathway not only restore the sensitivity of tumor cell growth but also increase the sensitivity of the tumor to chemotherapy, radiotherapy and hormone therapy.^235^ Moreover, cell cycle arrest was observed after suppression of the PI3K/Akt pathway.^236^ A recent study revealed that buparlisib, a class I PI3K inhibitor, could be safely combined with radiotherapy and improved the radiotherapeutic outcomes in patients with NSCLC.^237^ In conclusion, the combination of PI3K/Akt signaling pathway inhibitors and radiotherapy is a promising approach for cancer treatment.

#### Combination with immunotherapy

Although cancer is a genetic disease characterized by abnormal cell proliferation, it is also a chronic immune disease.^238^ Immunotherapy has recently attracted increasing attention and has indeed achieved some progress in treating cancer.^238^ Studies on the PI3K/Akt signaling pathway in immune cells have shown that its activation is not based on a simple on/off mechanism^239^; as cancer is a chronic immune disease, immunotherapies should be designed to increase the ability of immune cells to kill tumor cells such that the body exerts a direct antitumor effect. Determining whether drugs targeting the PI3K signaling pathway enhance or inhibit the efficacies of emerging immunotherapies will be critical for determining whether they can be combined. Both PI3K and mTOR inhibitors have been shown to enhance the efficacies of targeted immunotherapies in mouse tumor xenotransplantation models.^240^

Traditional adoptive cell therapy (ACT) is performed by isolating tumor-infiltrating lymphocytes (TILs) from biopsy materials.^241^ One of the main characteristics of TILs is their long-term persistence after migration. However, a major difficulty is that most TILs isolated from tumor tissues are terminally differentiated and do not form phenotypes with memory ability.^187^ Recently, ATC technology has been improved to enable the in vitro transduction of CD8+ T cells by the chimeric antigen receptor (CAR), with which the specific treatment of tumor antigens is not limited by major histocompatibility complex I.^242^ Recent reports on the efficacy of CAR-T cells for solid tumors in vivo indicate that it can be improved by PI3K inhibitors; however, these findings are preliminary, and the underlying mechanisms remain to be further elucidated.^242^

## Conclusions and remarks

In the past few decades, numerous insights into the PI3K/Akt signaling pathway have revealed its complex networks, including its mechanism of activation, upstream and downstream targets, and types of inhibitors, thereby increasing our understanding of the occurrence and development of different types of human cancers.

The diverse functions of PI3K/Akt signaling stem from downstream factors that link cellular function to stimulated upstream factors. For instance, extracellular growth factors recruit PI3K to the cell membrane, and the PI3K p110 subunit then induces the phosphorylation of PIP2 to PIP3, which promotes the localization of Akt to the plasma membrane. After activation by PDK1 and mTORC2, Akt drives the expression of targets associated with the cell phenotype. A dynamic system exists that comprises many substrates and crosstalk among them as well as crosstalk with other major signaling networks. This system exerts significant effects on modulating the oncogenicity of cancer.

After years of basic research, the development of inhibitors has undeniably advanced the treatment of cancer, but problems still persist. The most common problems related to inhibitors is their toxicity and side effects, such as hyperglycemia, rashes and other symptoms, and these effects on patients must not be ignored. In addition, concerns about the efficacy of animal models for predicting drug toxicity and whether in vivo toxicity can be predicted by in vitro experiments have been noted. Another problem is that conventional models, including phase I, II, and III trials, may not be suitable for testing PI3K inhibitors, which is detrimental to the determination of therapeutic efficacy. Although none of the inhibitors have entered phase III clinical trials, they show promise in treating cancer.^243^

Fortunately, most drugs have short half-lives and are easily managed, enabling early intervention; thus, patients treated with inhibitors can undergo active and effective intervention while monitoring for early toxicity identifiers. This pathway can be effectively manipulated to treat various human diseases caused by PI3K/Akt signaling dysregulation only when researchers fully elucidate the underlying mechanisms.
